# Editorial: The interaction between food ingredients and gut microbiome on health and disease

**DOI:** 10.3389/fmicb.2026.1809622

**Published:** 2026-03-27

**Authors:** Guiguo Zhang, Mohammad Altamimi, Junling Shi, Yunkyoung Lee

**Affiliations:** 1 Department of Animal Nutrition, Shandong Agricultural University, Taian, China; 2 China-South Korea International Joint Laboratory of Functional Polysaccharides in Shandong Province, Taian, China; 3 Department of Nutrition and Food Technology, Faculty of Agriculture Engineering and Veterinary Medicine, An-Najah National University, Nablus, Palestine; 4 School of Life Sciences, Northwestern Polytechnical University, Xi'an, China; 5 Department of Food Science and Nutrition, Jeju National University, Jeju, Republic of Korea

**Keywords:** disease, food ingredients, gut microbiome, interaction, metabolites

## Introduction

The intricate and dynamic ecosystem of microorganisms residing in the human gastrointestinal tract, collectively known as the gut microbiome, has emerged as a central player in human health and disease (Ley et al., 2006). This complex community, comprising trillions of bacteria, archaea, viruses, and fungi, does not merely exist in a passive commensal relationship with the host (Guo et al., 2025). Instead, it engages in a continuous, bidirectional crosstalk, profoundly influencing host physiology, metabolism, and immunity (Li et al., 2021; Liu et al., 2023). Among the myriad factors shaping the composition and function of the gut microbiome, diet stands out as one of the most potent and modifiable (Koh et al., 2016; Sonnenburg and Bäckhed, 2016).

The specific food ingredients consumed by host act as both substrates and signals for the gut microbiota, thereby modulating the production of a vast array of microbial metabolites (Zmora et al., 2019). These metabolites, including short-chain fatty acids (SCFAs), bile acids, vitamins, and neurotransmitters, serve as key messengers in the gut-host axis, impacting systems far beyond the intestines, from hepatic function to cerebral health (Koh et al., 2016; Li et al., 2021). The Research Topic “*The Interaction Between Food Ingredients and Gut Microbiome on Health and Disease*” brings together a compelling Research Topic of original research and reviews that delve deep into this critical interaction, exploring how diverse dietary components, from traditional herbs and prebiotics to food contaminants, can be leveraged to promote health or, conversely, contribute to pathophysiology.

The growing body of evidence reveals that dietary interventions targeting the gut microbiome hold immense promise for preventing and treating a spectrum of conditions, including metabolic disorders, inflammatory diseases, and even neurological conditions (Zhang et al., 2025; Zhao et al., 2020). This paradigm shift toward microbiome-targeted therapeutics is fueled by the understanding that dietary precision, moving beyond one-size-fits-all recommendations, could be the future of nutritional science (Levé et al., 2025; Zeevi et al., 2015). However, unraveling the precise mechanisms underlying the diet-microbiome-host interaction is a formidable challenge, necessitating a multidisciplinary approach that integrates microbiology, metabolomics, immunology, and bioinformatics (Choi et al., 2021; Wali et al., 2023).

This Research Topic serves as a testament to these efforts, presenting cutting-edge research that moves from correlation to causation, employing sophisticated models to dissect the molecular pathways involved. The findings highlighted in this issue not only reinforce the established roles of dietary fibers and fermented foods but also shed light on the potential of underutilized botanical extracts and the risks posed by modern environmental pollutants, thereby painting a more comprehensive picture of our dietary landscape's impact on gut ecological stability. Furthermore, the concept of personalized nutrition, driven by an individual's unique microbial blueprint, is gaining traction, suggesting that the efficacy of a food ingredient is inherently tied to the pre-existing gut microbial community structure. This editorial introduces and contextualizes the 36 articles (8 reviews and 28 original research articles) that form the cornerstone of this topical Research Topic, each contributing a vital piece to the complex puzzle of how what we eat dictates who we are, through the lens of our gut microbes.

Our synthesis of the published contributions reveals clear research trends while also underscoring persistent gaps in the field. The dominant themes spans dietary fibers/polysaccharides, polyphenols and botanicals, probiotics and postbiotics, broader dietary patterns, and macronutrients, environmental, or foodborne exposures, alongside several integrative reviews. Across these studies, commonly reported readouts of microbiome modulation include SCFAs, inflammatory markers, gut barrier integrity, immune, and neurological endpoints, and metabolic indicators.

Despite this progress, important gaps remain, most notably in methodological standardization and transparent reporting. Moreover, relatively few studies establish causal, mechanistic links between specific dietary components, microbiome remodeling, and downstream host physiology (Martens et al., 2011; Zhao et al., 2018). Although multi-omics designs are increasingly used, proposed connections among microbial shifts, metabolite changes, and clinical or metabolic improvements often remain inferential (Li et al., 2026; Wang et al., 2024). Strengthening causal inference will require functional validation (e.g., fecal microbiota transplantation (FMT), germ-free/gnotobiotic models, and targeted intervention experiments), so that frequently invoked frameworks such as the gut-liver axis move from plausible models to demonstrated mechanisms.

## Summary of articles published in the Research Topic

This Research Topic brings together 36 contributions examining the dynamic interplay among dietary factors, gut microbial communities, and host health. The Research Topic spans animal experiments, human clinical work, and comprehensive reviews, highlighting the gut microbiome as a central mediator in metabolic, neurological, inflammatory, and systemic conditions. Below, we briefly outline each of the 36 papers, noting the focal ingredient or intervention, the main outcomes, and the broader relevance of findings.

Animal experiments collectively underscore the therapeutic promise of diverse ingredients and strategies. Work on plant polyphenols reported that both proanthocyanidins and rutin improved type 2 diabetes in mice, with proanthocyanidins showing greater efficacy, accompanied by distinct gut microbiota shifts (Gao et al.). Likewise, flavonoids from *Amomum tsaoko* (ATF) mitigated ulcerative colitis by suppressing inflammatory signaling and enriching beneficial taxa such as *Akkermansia, Bifidobacterium and* unclassified_f_*Atopobiaceae*, via the gut microbiota-LPS/TLR4/NF-κB/NLRP3 axis (Huang et al.). In a related botanical study, an extract from *Amomum tsao-ko Crevost et Lemarie* attenuated atherosclerosis by influencing the gut-liver axis, lowering serum cholesterol, reshaping the gut microbial composition, and reducing systemic inflammation and oxidative stress (Wang Q. et al.). Additional plant-derived interventions were also effective: *Paederia scandens* extract countered obesity-associated dysbiosis by restoring microbial diversity and shifting serum metabolites linked to amino acid and energy metabolism (Yang Y. et al.). In livestock, *Codonopsis pilosula* polysaccharides supported intestinal health and immunity in Hu sheep, enhancing microbial diversity and upregulating gut barrier proteins (Zhao Q. et al.). Furthermore, ferulic acid and its derivative N-feruloylserotonin reduced intestinal inflammation through a multi-omics mechanism involving microbiota remodeling, enrichment of beneficial metabolites (e.g., 1-naphthalenesulfonic acid), and regulation of NF-κB and MAPK signaling (Hu et al.). Traditional formulas further broadened the mechanistic landscape. *Dachaihu* decoction improved autism spectrum disorder-related outcomes in rats and in a human trial, consistent with gut-brain axis modulation, including enrichment of beneficial taxa (e.g., *Adlercreutzia*), reduced inflammation and oxidative stress, and improved barrier integrity (Zhang, Li et al.). Separately, Guiren Runchang Granules (GRG) improved slow transit constipation by rebalancing gut microbial ecology, increasing SCFA production, and normalizing motility-related hormones (e.g., serotonin and motilin) (Bai et al.).

Probiotic, postbiotic, and prebiotic approaches featured prominently across multiple disease settings. Notably, pasteurized *Akkermansia muciniphila* (a postbiotic) outperformed the live strain in murine colitis model, strengthening barrier function and dampening inflammation (Han et al.). For metabolic outcomes, *Clostridium butyricum* improved obesity and glucose intolerance by restoring barrier integrity, reducing systemic inflammation, and lowering microbially derived branched-chain amino acids (Zhang X. et al.). In poultry, *Bacteroides uniformis* counteracted diet-induced fatty liver disease in laying hens by reconfiguring the gut microbiome and correcting arachidonic acid metabolism, thereby reducing hepatic lipid deposition (Zhang, Ma et al.). A separate study addressed environmental toxicants, showing that *Lactiplantibacillus plantarum* ZP-6 lessened nanoplastic-induced liver and colon damage by promoting fecal excretion (via adsorption) and reinforcing the intestinal barrier through the gut-liver axis (Zhao L. et al.). Dietary substrates were likewise informative. Galacto-oligosaccharides (GOS) improved lactose intolerance in mice in a microbiota-dependent manner, enriching lactose-metabolizing bacteria (notably *Lactobacillus* and *Bifidobacterium*), enhancing barrier function, and reducing inflammation (Li Q. et al.). In contrast, an evaluation of sweeteners found that synthetic compounds (sucralose and saccharin) substantially reduced microbial diversity and enriched potential pathogens (e.g., *Enterobacteriaceae*), whereas non-synthetic alternatives (Rebaudioside A and xylitol) were less disruptive and in some cases strengthened beneficial microbial networks (Kidangathazhe et al.).

Micronutrients and supplements were also explored. In a neurodevelopmental framework, dietary zinc improved autism-like behaviors in Shank3B-/- mice by reshaping bacterial and fungal communities and affecting host genes involved in metabolism and microbe-host interactions (Wong et al.). In ruminant nutrition, L-citrulline enhanced reproductive performance (estrus rate) in Hu ewes through rumen microbiota shifts, improved antioxidant capacity, and stimulation of reproductive hormone secretion (Liu et al.). A blend of medium- and short-chain fatty acids (MCFA/SCFA) promoted gut health in weaned piglets as a zinc oxide alternative by reducing diarrhea, improving antioxidant status, and enriching beneficial taxa such as *Lactobacillus* and *Roseburia* (Fan et al.). Another phytochemical intervention, chive seed flavonoid improved intestinal morphology, increased volatile fatty acid production, and expanded microbial diversity in sheep, consistent with enhanced gut development and function (Li X. et al.). Extending beyond intestinal outcomes, a chewable tablet containing rapeseed bee pollen and maca alleviated benign prostatic hyperplasia in rats by restoring microbiota balance and inhibiting IL-6/JAK2/STAT3 signaling, demonstrating a gut-organ axis mechanism (Huang et al.).

Work on ecological and pathological interactions clarified how diet, management, and infection shape gut communities in animal systems. An intervention of arid-adapted rodents indicated that host phylogeny and dietary composition were key drivers of gut community structure, with plant-based foods enhancing microbial diversity and animal-based consumption reducing it (Cha et al.). In goats, husbandry-related drivers were also evident. One report showed how high dietary energy and protein shifted cecal fermentation, increased microbial diversity, and altered host gene expression linked to nutrient absorption and intestinal immunity (Fu et al.). Another study associated Caprine Arthritis Encephalitis virus infection with distinct fecal microbiome signatures and identified intensive farming (relative to pasture-based systems) as an additional factor influencing microbial composition (Molotzu et al.).

*In vitro* platforms provided higher-resolution views of microbiome dynamics. One study delineated how *Aloe vera* and dandelion extracts exert prebiotic effects, such as enriching *Akkermansia muciniphila* and *Bifidobacterium*, in a taxon-dependent manner shaped by cross-feeding networks involving key members such as *Bacteroides* spp. (Mancabelli et al.). Another *in vitro* model tracked the fate of the foodborne pathogen *Listeria monocytogenes*, showing that clinical isolates were more resistant to simulated digestion than food-borne strains and that infection reconstructed the microbiota, increasing ethanol-producing groups (Kim et al.). Bridging models systems and environmental health, a translational analysis proposed a gut-vascular axis for bisphenol F (BPF), demonstrating in patients and rats that BPF exposure promotes vascular calcification via dysbiosis and downstream inflammatory responses (Yan et al.).

Human clinical studies added findings with direct translational relevance. In colorectal adenoma (CRA) progression, stage-specific microbial patterns were observed, including depletion of protective, SCFA-producing genera (e.g., *Anaerostipes* and *Blautia*) in advanced lesions, alongside a progressive rise in PD-L1 pointing to candidate microbiome-based biomarkers for earlier detection (Wang X. et al.). Complementing this, an *in vitro* nutritional experiment suggested that microbial responses to foods are shaped less by single nutrients than macronutrient balance; pulses such as red and mung beans (with more favorable ratio) most strongly supported eubiosis and reduced pathobionts such as *Escherichia-Shigella* (Lee and Hwang). Consistent with individualized responsiveness, a personalized nutrition trial reported that microbiota-tailored granola increased beneficial taxa (including *Bifidobacterium*), enhanced SCFA production, and was associated with measurable improvements in mood among participants (Sasaki et al.).

In addition to the original articles, eight reviews synthesized broader evidence across diverse contexts, each examining how diet-microbiome interactions intersect with specific disease pathways. One narrative review consolidated evidence on lifestyle factors, diet, sleep, and exercise, showing that high-fiber diets and regular physical activity support beneficial SCFA-producing communities, whereas circadian disruption favors pro-inflammatory shifts and reduced microbial diversity (Zeng et al.). Another review assessed mechanistic links between high-fat diets, microbiota disruption, and precocious puberty, highlighting potential roles for microbial metabolites in endocrine signaling (Wu et al.). A further synthesis discussed the gut-brain axis in Alzheimer's disease, positioning diet as a modifiable determinant in pathogenesis (Siripaopradit et al., 2024). Beyond neurological outcomes, one review described how microbiota-derived metabolites (including SCFAs and bile acids) may contribute to vitiligo through gut-skin axis signaling and immune modulation (Yuan et al.). Shifting to animal health, another review examined the equine gut microbiome, detailing how diet and management practices influence its composition and function (Li F. et al.). From a clinical perspective, one article summarized microbiota-directed strategies, probiotics, FMT, and bacteriophage therapy, for alleviating diarrhea by re-establishing microbial balance (Tian et al.). Finally, a review highlighted beneficial taxa and dietary patterns that promote health, including recent advances in personalized nutrition and AI-enabled approaches for microbiome modulation (Kumar et al.). Collectively, these reviews offer a multi-faceted perspective on how dietary factors engage the gut microbiome to influence health and disease across species and life stages.

## Critical appraisal and future directions

The collective works presented in this Research Topic represent a significant leap forward in our mechanistic understanding of the diet-gut microbiome axis. The research moves beyond simple correlations, employing germ-free models, gnotobiotic animals, sophisticated *in-vitro* fermentation systems, and multi-omics integration to establish causality and elucidate pathways. The consistent theme across numerous studies is the potent efficacy of plant-derived compounds (polyphenols, flavonoids, and polysaccharides from herbs like Amomum tsao-ko, Codonopsis pilosula, and Paederia scandens) in ameliorating conditions from metabolic syndrome to neuroinflammation, primarily through microbial modulation. This reinforces the immense value of exploring traditional pharmacopeias for novel microbiome-targeting therapies. Furthermore, the studies on personalized nutrition and the strain-specific effects of botanical extracts powerfully underscore a critical paradigm: the host's baseline microbiota is a key determinant of nutritional response, pushing the field toward personalized approaches.

Despite these advances, several challenges and opportunities for future research remain. First, while animal models are indispensable, there is a pressing need for more long-term, well-controlled human intervention studies to validate these findings and determine effective doses for human application. Second, the sheer complexity of the interactions, where a single dietary component can simultaneously alter dozens of microbial taxa and host pathways, calls for more sophisticated computational and systems biology approaches to move from descriptive lists to predictive models of host-microbiome dynamics. A notable gap in the current Research Topic, and the field at large, is the limited exploration of non-bacterial members of the microbiome, such as fungi (the mycobiome), viruses (the virome), and archaea. Their roles in mediating the effects of diet are likely substantial but remain underexplored.

Future research should also focus on the temporal dynamics of microbial responses to dietary interventions. Understanding the resilience of the microbiome and the sustainability of dietary-induced changes is crucial for developing long-term strategies (Ko et al., 2025; Lin et al., 2025). Finally, translating this knowledge into public health and clinical practice requires the development of robust biomarkers of microbiome health and the integration of microbiome data into dietary guidelines- a formidable but essential task for the future of precision nutrition (Mann et al., 2024; Rondinella et al., 2026).

In conclusion, this Research Topic successfully captures the vibrancy and depth of contemporary research on the interaction between food ingredients and the gut microbiome. The collected articles provide compelling evidence that dietary manipulation of the gut microbiota is a powerful and viable strategy for promoting health and combating disease. From elucidating the mechanisms of ancient herbs to highlighting the risks of modern pollutants and pioneering personalized nutrition, the work presented here charts a course for future discovery. The key takeaway is that the future of nutritional science lies in embracing complexity-acknowledging the individuality of our microbial partners, the multi-kingdom nature of the gut ecosystem, and the dynamic interplay between diet, microbes, and host physiology. The insights garnered from this Research Topic firmly establish the gut microbiome not as a passive passenger, but as a central mediator of dietary health effects, opening exciting new avenues for therapeutic innovation.

## References

[B1] ChoiB. S. Y. DanielN. HoudeV. P. OuelletteA. MarcotteB. VarinT. V. . (2021). Feeding diversified protein sources exacerbates hepatic insulin resistance via increased gut microbial branched-chain fatty acids and mTORC1 signaling in obese mice. Nat. Commun. 12:3377. doi: 10.1038/s41467-021-23782-w34099716 PMC8184893

[B2] GuoC. MaD. ZhangC. WangY. UllahF. WangX. . (2025). Dietary Jerusalem artichoke polysaccharide supplementation alters the growth performance, ruminal microbes and metabolites, muscle fatty acid and amino acid profiles in fattening lambs. Anim. Nutr. 22, 139–153. doi: 10.1016/j.aninu.2025.02.00740843217 PMC12365176

[B3] Ko K. Guo C. Kim E. Zhang G. Lee Y. (2025). Structural features of galacturonic acid-rich polysaccharides from Ziziphus jujuba and their protective effects against insulin resistance and muscle atrophy. Int. J. Biol. Macromol. 322:146287. doi: 10.1016/j.ijbiomac.2025.146287 40782857

[B4] Koh A. De Vadder F. Kovatcheva-Datchary P. Bäckhed F. (2016). From dietary fiber to host physiology: short-chain fatty acids as key bacterial metabolites. Cell 165, 1332–1345. doi: 10.1016/j.cell.2016.05.041 27259147

[B5] LevéM. ManghiP. BredonM. LefevreA. ManaraS. ArmaniniF. . (2025). Metabolomics and metagenomics in mice reveal the role of the gut microbiota in tryptophan metabolism. iScience 28:113751. doi: 10.1016/j.isci.2025.11375141234773 PMC12606010

[B6] Ley R. E. Turnbaugh P. J. Klein S. Gordon J. I. (2006). Microbial ecology: human gut microbes associated with obesity. Nature 444, 1022–1023. doi: 10.1038/4441022a 17183309

[B7] LiD. FengY. TianM. JiJ. HuX. ChenF. (2021). Gut microbiota-derived inosine from dietary barley leaf supplementation attenuates colitis through PPARγ signaling activation. Microbiome 9:83. doi: 10.1186/s40168-021-01028-733820558 PMC8022418

[B8] Li Z. Kang M. Ko K. Song Y. Lee Y. Zhang G. (2026). Dietary inulin improves growth performance and systemic health of fattening goats by modulating rumen microbiome and metabolome. Anim. Feed Sci. Technol. 334:116641. doi: 10.1016/j.anifeedsci.2026.116641

[B9] Lin Y. Wu J. Huang H. Zeng Z. Zhang W. Wang L. . (2025). Mechanisms of Laminaria japonica polysaccharide digestion and absorption: structure, in vivo fluorescence imaging, and gut microbiota. J. Agric. Food Chem. 73, 21148–21161. doi: 10.1021/acs.jafc.5c03896 40790025

[B10] LiuA. KimE. CuiJ. LiJ. LeeY. ZhangG. (2023). Laminaria japonica polysaccharide improved the productivities and systemic health of ducks by mediating the gut microbiota and metabolome. J. Agric. Food Chem. 71, 7382–7395. doi: 10.1021/acs.jafc.2c0873137150978 PMC10197123

[B11] Mann E. R. Lam Y. K. Uhlig H. H. (2024). Short-chain fatty acids: linking diet, the microbiome and immunity. Nature Rev. Immunol. 24, 577–595. doi: 10.1038/s41577-024-01014-8 38565643

[B12] MartensE. C. LoweE. C. ChiangH. PudloN. A. WuM. McNultyN. P. . (2011). Recognition and degradation of plant cell wall polysaccharides by two human gut symbionts. PLoS Biol. 9:e1001221. doi: 10.1371/journal.pbio.100122122205877 PMC3243724

[B13] Rondinella D. Margarita E. Raoul P. C. Galli F. S. Severino A. Porcari S. . (2026). The impact of diet on gut microbiome composition: implications for immune-mediated diseases. Clin. Immunol. Commun. 9, 1–11. doi: 10.1016/j.clicom.2025.12.001

[B14] SiripaopraditY. ChatsirisakulO. AriyapaisalkulT. SereemaspunA. (2024). Exploring the gut-brain axis in Alzheimer's disease treatment via probiotics: evidence from animal studies-a systematic review and meta-analysis. BMC Neurol. 24:481. doi: 10.1186/s12883-024-03978-539695988 PMC11657461

[B15] SonnenburgJ. L. BäckhedF. (2016). Diet–microbiota interactions as moderators of human metabolism. Nature 535, 56–64. doi: 10.1038/nature1884627383980 PMC5991619

[B16] WaliJ. A. NiD. FaceyH. J. W. DodgsonT. PulpitelT. J. SeniorA. M. . (2023). Determining the metabolic effects of dietary fat, sugars and fat-sugar interaction using nutritional geometry in a dietary challenge study with male mice. Nat. Commun. 14:4409. doi: 10.1038/s41467-023-40039-w37479702 PMC10362033

[B17] Wang K. Zhou Y. Li M. Chen Z. Wu Z. Ji W. . (2024). Structural elucidation and immunomodulatory activities in vitro of type I and II arabinogalactans from different origins of Astragalus membranaceus. Carbohydr. Polym. 333:121974. doi: 10.1016/j.carbpol.2024.121974 38494227

[B18] Zeevi D. Korem T. Zmora N. Israeli D. Rothschild D. Weinberger A. . (2015). Personalized nutrition by prediction of glycemic responses. Cell 163, 1079–1094. doi: 10.1016/j.cell.2015.11.001 26590418

[B19] ZhangL. Marfil-SánchezA. KuoT.-H. SeelbinderB. van DamL. Depetris-ChauvinA. . (2025). Gut microbiome-mediated transformation of dietary phytonutrients is associated with health outcomes. Nat. Microbiol. 11, 94–110. doi: 10.1038/s41564-025-02197-z41339745 PMC12768975

[B20] Zhao F. Liu Q. Cao J. Xu Y. Pei Z. Fan H. . (2020). A sea cucumber (*Holothuria leucospilota*) polysaccharide improves the gut microbiome to alleviate the symptoms of type 2 diabetes mellitus in Goto-Kakizaki rats. Food Chem. Toxicol. 135:110886. doi: 10.1016/j.fct.2019.110886 31626838

[B21] Zhao L. Zhang F. Ding X. Wu G. Lam Y. Y. Wang X. . (2018). Gut bacteria selectively promoted by dietary fibers alleviate type 2 diabetes. Science 359, 1151–1156. doi: 10.1126/science.aao5774 29590046

[B22] Zmora N. Suez J. Elinav E. (2019). You are what you eat: diet, health and the gut microbiota. Nat. Rev. Gastro Hepat. 16, 35–56. doi: 10.1038/s41575-018-0061-2 30262901

